# Control of fluid balance guided by body composition monitoring in patients on peritoneal dialysis (COMPASS): study protocol for a randomized controlled trial

**DOI:** 10.1186/1745-6215-15-432

**Published:** 2014-11-06

**Authors:** Seon Ha Baek, Kook-Hwan Oh, Sejoong Kim, Dong Ki Kim, Kwon-Wook Joo, Yun Kyu Oh, Byoung Geun Han, Jae Hyun Chang, Wookyung Chung, Yon Su Kim, Ki Young Na

**Affiliations:** Department of Internal Medicine, Seoul National University Bundang Hospital, 82, Gumi-ro 173 Beon-gil, Bundang-gu, Seongnam, 463-707 Gyeonggi-do South Korea; Seoul National University Hospital, Seoul, South Korea; Seoul National University Boramae Medical Center, Seoul, South Korea; Wonju Severance Christian Hospital, Wonju, South Korea; Gachon University Gil Hospital, Incheon, South Korea

**Keywords:** Fluid balance, Bioimpedance spectroscopy, Peritoneal dialysis

## Abstract

**Background:**

The clinical benefits of bioimpedance spectroscopy (BIS)-guided fluid management in patients on hemodialysis have been widely demonstrated. However, no previous reports have evaluated the effect of regular and serial BIS-guided fluid management on the residual renal function (RRF) in patients on peritoneal dialysis (PD). Therefore, we will evaluate the clinical efficacy of BIS-guided fluid management for preserving RRF and protecting cardiovascular events in patients on PD.

**Methods/design:**

This is a multicenter, prospective, randomized controlled trial. A total of 138 participants on PD will be enrolled and randomly assigned to receive either BIS-guided fluid management or fluid management based only on the clinical information for 1 year. The primary outcome is the change in the glomerular filtration rate (GFR) between months 0 and 12 after starting treatment. The secondary outcomes will include GFR at month 12, time to the anuric state (urine volume <100 ml/day), and fatal and nonfatal cardiovascular events during treatment.

**Discussion:**

This is the first clinical trial to investigate the effect of BIS-guided fluid management on RRF and for protecting against cardiovascular events in patients on PD.

**Trial registration:**

Clinical Trials.gov number NCT01887262, June 24, 2013.

## Background

Overhydration (OH) is common, and is associated with cardiac dysfunction and mortality in patients on peritoneal dialysis (PD) [1, 2]. By contrast, volume depletion is associated with a more rapid loss of residual renal function (RRF) in patients on PD [3]. Therefore, euvolemia is one of the prime objectives in these patients.

In clinical practice, the volume status is indirectly assessed using various clinical data, including edema, weight gain, and hypertension [4]. Although there is a linear relationship between blood pressure (BP) and tissue hydration, a substantial proportion of patients do not follow that pattern. A number of patients who are euvolemic or underhydrated have systolic hypertension. Volume reduction to control systolic hypertension may result in dehydration in these patients, which may decrease RRF or compromise coronary artery perfusion, and may result in acute myocardial infarction [5]. Conversely, patients with congestive heart failure may have a low or normal BP despite OH. Therefore, BP cannot reflect changes in the hydration status [2].

Bioimpedance spectroscopy (BIS) has been used to measure the water compartment of the body [6]. In patients on dialysis, body composition, including extracellular water (ECW) and intracellular water (ICW), muscle mass, and fat mass, changes slowly over several months. Regular monitoring of body composition using BIS can facilitate the optimal management of fluid balance in patients on dialysis.

The purpose of the present study is to evaluate the clinical usefulness of BIS-guided fluid management for preserving RRF and protecting against cardiovascular events in patients on PD.

## Methods/design

### Hypothesis

BIS-guided fluid management will preserve RRF in patients on PD better than fluid management guided by clinical information alone, such as BP, body weight (BWT), and physical examination results.

### Study design

**Figure 1:**
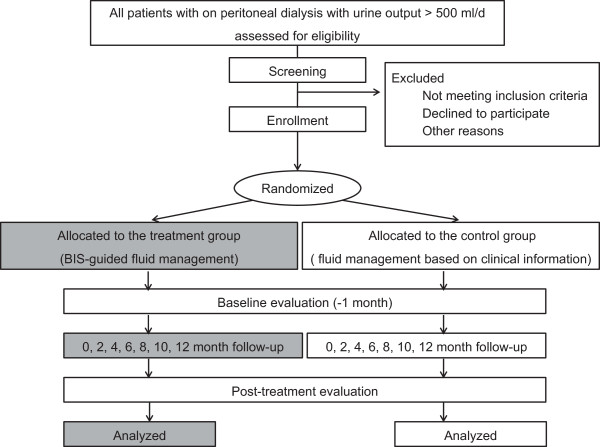
**Study algorithm.** BCM, body composition monitoring.

The study is a multicenter, prospective, parallel-group, open-label, randomized controlled trial. It is an investigator-initiated clinical trial. The study algorithm is depicted in Figure 1. After enrollment, clinical follow-up will be performed after 0, 2, 4, 6, 8, 10, and 12 months of treatment.

### Study participants and measurements

All patients will be selected from the outpatient renal clinic of five tertiary hospitals in Korea (Seoul National University Bundang Hospital, Seoul National University Hospital, Seoul National University Boramae Medical Center, Gachon University Gil Hospital, and Wonju Severance Christian Hospital). Patients aged 20 to 75 years who are on PD and have a daily urine output of more than 500 ml will be screened [7]. The following tasks will be conducted at the initial visit: 1) completion of questionnaire on medical and drug history, including the use of anti-hypertensive medication and diuretics; 2) physical examination of all body systems; 3) measurement of height and weight with an empty abdomen; and 4) BP and pulse rate measurement. Participants who meet all of the inclusion and exclusion criteria and who provide written informed consent will be eligible for this study.

### Inclusion and exclusion criteria

Inclusion criteria were: age between 20 and 75 years; duration of PD (automated peritoneal dialysis (APD) or continuous ambulatory peritoneal dialysis (CAPD)) >4 weeks; daily urine output >500 ml; and provision of written informed consent.

Exclusion criteria were: contraindication to bioimpedance measurement (amputation, presence of pacemaker, defibrillator, prosthesis, or metal implants); probable discontinuation of PD or receipt of kidney transplant within 1 year; hypoalbuminemia (serum albumin <3.3 g/dl); severe heart failure (New York Heart Association Functional Classification (NYHA FC) III or IV); combined dialysis modality (PD + intermittent hemodialysis (HD)); pregnancy, lactation; enrollment in other clinical trials within 1 month; uncontrolled hypertension (>160/100 mmHg with more than three anti-hypertensive medications); cardiovascular diseases (cerebral infarction, hemorrhagic infarction, acute myocardial infarction, or unstable angina) and acute infection (pneumonia, peritonitis) within 3 months prior to the trial.

Serum and urine creatinine (Cr) will be measured by the isotope dilution mass spectrometry-traceable method using a TBA 200FR Analyzer (Toshiba, Tokyo, Japan). GFR will be calculated as the average Cr and urea clearance, which is measured by urine collection [8].

### Randomization

The randomization process will be conducted using a web-based computer program. A list of random numbers will be generated by a computerized random allocation system operated by the Medical Statistics Support Team in Seoul National University Hospital. Eligible participants will be randomly assigned 1:1 to either the control group (fluid management based on the clinical information alone) or treatment group (BIS-guided fluid management along with clinical information). Randomization will be stratified based on the institution and presence of diabetes mellitus, and will utilize a randomized block design.

### Outcome measures

The primary outcome is the change in GFR between months 0 and 12 after starting treatment. The secondary outcomes will include GFR measured by urine collection, calculated by the mean of the Cr and urea clearance at month 12; time to the anuric state (urine volume <100 ml/day); and fatal and nonfatal cardiovascular events, including acute myocardial infarction, stroke, unstable angina, and cardiovascular revascularization. Parameters obtained by echocardiographic measurements, such as the left ventricular mass index, E/e′ ratio, left ventricular end-diastolic pressure, left ventricular ejection fraction, left atrial volume index, and BP at month 12; parameters measured by BIS, such as the OH value, ECW, and ECW/ICW; hospitalization, cardiovascular and all-cause mortality, and transfer to HD over the course of 1 year; pulse wave velocity (PWV), type and number of PD fluid and diuretics at month 12, and laboratory findings including high-sensitivity C-reactive protein (hs-CRP), N-terminal prohormone of brain natriuretic peptide, and cardiac troponin T, will also be evaluated as tertiary outcomes.

### Assessment of the fluid status

For participants in the control group, clinical information obtained by physician physical examination is the standard of judgment. Clinical information is composed of four items, including measured BWT within 2 kg of the dry weight, BP less than 140/90 mmHg, absence of symptoms and signs for hypervolemia (dyspnea, edema, and crackle) or hypovolemia (dizziness and orthostatic hypotension).

For participants in the treatment group, the OH value measured by the Body Composition Monitor (BCM) (Fresenius, Medical Care Korea, Seoul, Korea), using an alternating current at 50 different frequencies (5–1000 kHz) combined with clinical information, represents the standard of judgment. The OH value is calculated using the BCM based on a physiologic tissue model that is composed of the individual’s normal ECW, normohydrated lean and adipose tissue. The OH value can be calculated from the difference between the normal expected ECW and the measured ECW [9]. The BCM has been validated with isotope dilution methods against all available gold-standard methods in the healthy population and in patients on dialysis [10–12].

### Physician’s practical treatment guidelines according to the volume status

**Figure 2:**
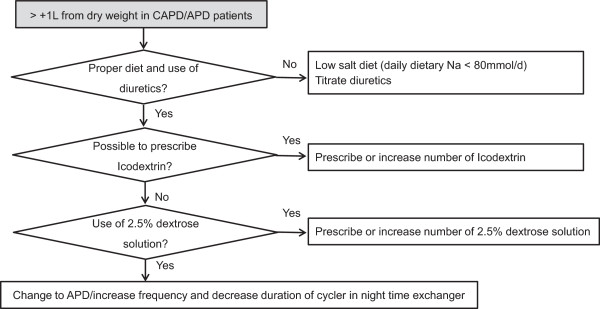
**Hypervolemia management flow chart.** APD, automated peritoneal dialysis; CAPD, continuous ambulatory peritoneal dialysis.

**Figure 3:**
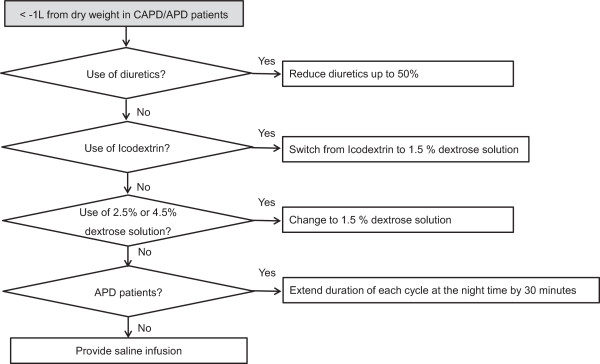
**Hypovolemia management flow chart.** APD, automated peritoneal dialysis; CAPD, continuous ambulatory peritoneal dialysis.

The target BWT will be within 1 liter of the dry BWT. After randomization, participants in the control group will undergo BCM measurement at the beginning and end of the study. Both the physicians and participants will be blinded to the results. The physicians will prescribe PD solutions, drugs, and diet based on the clinical information that they obtain. Participants in the treatment group will undergo BCM measurement every 2 months over a 1-year period. Both the physicians and the participants will be notified of the results. Based on the BCM results combined with the clinical information, the physicians will prescribe PD solutions, diuretics, and diets. Figures 2 and 3 show the management of hypervolemia and hypovolemia, respectively.

Participants who switch to HD or combined dialysis (PD with intermittent HD), receive kidney transplantation, transfer to other institutions, or die during the study will be dropped out of the study. Investigators or research coordinators will evaluate treatment adherence and give scores for each particular item, including 1) percentage of self-reported medical records >80%, 2) percentage receiving PD solutions >80%, 3) adherence to examinations including complete blood count (CBC), chemistry, peritoneal equilibrium test, Kt/V (where K is dialyzer clearance of urea, t is dialysis time, and V is volume of distribution of urea, approximately equal to the patient's total body water), and 4) adherence to the scheduled visits.

### Clinical and laboratory evaluations

Physical examination, medication reviews including PD solutions, fluid balance evaluation (semi-quantitative scale),. and laboratory evaluations, including CBC, electrolytes, Cr, protein, albumin, calcium, phosphorous, and fasting glucose will be conducted every 2 months.

Laboratory evaluations, including total cholesterol, triglyceride, low-density lipoprotein cholesterol, hs-CRP, hemoglobin A1C, intact parathyroid hormone, N-terminal prohormone of brain natriuretic peptide, cardiac troponin T, and weekly Kt/V_urea_, will be performed at months 0 and 12 of treatment. Echocardiography will be performed, and PWV will be measured using a Colin pulse waveform analyzer (Colin CO, Ltd, Komaki, Japan) at months 0 and 12 after treatment. The study schedule is shown in Table 1.

**Table 1:** **Data collection schedule of the COMPASS study**

Parameter	Visit time (months)							
	-1	0	2	4	6	8	10	12
Demographic information	X							
Charlson Comorbidity Index	X							
Physical examination^1^	X	X	X	X	X	X	X	X
Medication review^2^	X	X	X	X	X	X	X	X
Routine laboratory data^3^		X	X	X	X	X	X	X
Special laboratory data^4^		X						X
RRF, urine volume		X						X
Compliance		X	X	X	X	X	X	X
New events^5^		X	X	X	X	X	X	X
BCM measurement								
Treatment group		X	X	X	X	X	X	X
Control group		X						X
Kt/V		X						X
Peritoneal equilibration test		X						
PWV		X						X
Echocardiography		X						X
PD solution, medication change		X	X	X	X	X	X	X

BCM*,* Body composition monitoring; COMPASS*,* Control Of fluid balance guided by body composition Monitoring in patients on PeritoneAl dialySiS trial; Kt/V*,* Calculated using single pool equation; PD*,* Peritoneal dialysis; PWV*,* Pulse wave velocity; RRF*,* Residual renal function.

^1^Includes body weight with an empty abdomen, blood pressure, pulse rate, and edema.

^2^Number of anti-hypertensive medications and diuretics and the type of PD solution.

^3^Complete blood count, electrolyte, serum creatinine, protein, albumin, calcium, phosphorous, and fasting glucose.

^4^Total cholesterol, triglyceride, low-density lipoprotein cholesterol, high-sensitivity C-reactive protein, hemoglobin A1C, intact parathyroid hormone, N-terminal prohormone of brain natriuretic peptide, and cardiac troponin T.

^5^Any new clinical event, including hospitalization and peritonitis, will be recorded during the study period.

### Safety issues

The BIS method approved by the US Food and Drug Administration has been used in clinical practice, particularly in Europe. Weak alternating currents into the body are known to be not harmful.

### Sample size calculations

No previous report has evaluated the effect of BIS-guided fluid management on RRF in patients on PD. We therefore referred to a study showing that GFR changes over 1 year in a conventional PD solution treatment group and in a biocompatible PD solution treatment group were -16.8 ± 19.9 and 0.09 ± 40.9 l/week/1.73 m^2^, respectively [13]. We adjusted the sample size for an estimated drop-out rate of 20% due to poor compliance, a two-sided level of significance of α = 5%, and a power of 80%, and found that 69 participants will be required in each group to find a significant difference using Student’s *t*-test. A total of 138 participants will be included in the analysis.

### Statistical analyses

The statistical analyses will be conducted both on a per-protocol (PP) and an intention-to treat (ITT) basis. For PP analysis, all participants who complete the study will be included to evaluate the primary and secondary outcomes. For the ITT analysis, all participants who are enrolled and randomized to one of the two groups and who complete the first visit will be included.

Basic statistics will be reported in terms of mean ± SD for continuous variables, or as percentages for categorical variables. Differences between groups will be analyzed using Student’s *t*-test for continuous variables and the *χ*^2^ test or Fisher’s exact test for categorical variables. The difference in GFR between month 0 and month 12 will be compared between the two groups using Student’s *t*-test for the primary outcome. Analysis of covariance (ANCOVA) will be used to analyze the primary outcome as the secondary analysis to adjust the baseline value. Multivariate Cox proportional hazard regression models will be used to analyze the time to development of anuria. Although the institutions are mainly located in large cities, thereby limiting the likelihood of any important cluster effect, we will be sure to accommodate possible clustering in our models and analysis as required. A value of *P* < 0.05 will be considered statistically significant. All analyses will be performed using SPSS Statistics software (v21.0; IBM Corporation, Armonk, NY, USA).

### Ethics approval

The study will be performed in accordance with the Declaration of Helsinki, as amended by the 59th World Medical Association General Assembly in 2008. All the participants will provide signed, informed, written consent, stating that participation is voluntary and can be withdrawn at any time. Approval for the study has been obtained from the institutional review board of Seoul National University Bundang Hospital (E-1303/194-001), Seoul National University Hospital (H-1302-050-465), Seoul National University Boramae Medical Center (16-2013-30), Gachon University Gil Hospital (GAIRB2013-119), and Wonju Severance Christian Hospital (CR312065). The trial protocol has been registered at http://www.clinicaltrials.gov (NCT01887262).

## Discussion

Preservation of RRF has been shown to be associated with a reduction in mortality, and has become one of the prime objectives in the treatment of patients on PD [14, 15]. Controversy remains about whether hypervolemia or strict volume control helps preserve RRF in these patients [3, 16, 17]. Other groups have reported that BIS may be superior to clinical information in the assessment of fluid status for patients on HD or PD [2, 9, 12, 18]. However, no studies have evaluated the effect of regular and serial BIS-guided fluid management on RRF in patients receiving PD. To our knowledge, this is the first multicenter, prospective, randomized controlled trial assessing whether BIS-guided volume management attenuates the loss of RRF. The endpoints of the COMPASS (Control Of fluid balance guided by body composition Monitoring in patients on PeritoneAl dialySiS) study are of the utmost importance to healthcare providers.

The strength of the study is that only subjects who are on PD for more than 1 month are eligible. This criterion reduces the likelihood of a pre-dialytic uremic factor confounding the primary outcome. Potential covariates, such as, the differences in treatment strategies between physicians, can also be minimized because physicians will prescribe PD solutions, diuretics, and diet according to the same protocol. Therefore, it is reasonable that the type and number of PD solutions and diuretics should serve as the secondary outcomes.

The OH value has been widely used in the assessment of volume status in patients on PD [19–21]. Additionally, O’Lone et al. recently reported the clinical significance of the OH index, - an accurate value of overhydration that was an independent predictor of mortality in contrast to the ECW/total body water (TBW) [22]. Different BIS parameters, such as ECW/TBW, ECW normalized for height, the OH value, and tissue hydration, have been used as indices of hydration in previous studies [6, 9, 10]. In other words, there is no definite BIS parameter for assessing the hydration status. In this respect, the usefulness and relevance of BIS various parameters will be determined in the present study.

Based on normal hydration being defined as ECW within ±1.1 liter of the 10th to the 90th percentiles in healthy subjects [10], previous studies for patients on HD have applied the same criteria [23–25]. Although some studies have suggested cutoff thresholds for the definition of the OH in patients on HD and patients on PD of 2.5 and 2.0 liters, respectively [19, 26, 27], we consider an ECW within ±1.1 liter as normal hydration.

There is a chance that patients within the same institution will be more correlated than patients between institutions. To avoid this, we will investigate for possible clustering of patients at the recruitment institution level, and will accommodate such clustering in our models and analysis as required. We also conservatively calculate the sample size, assuming a high drop-out rate, to overcome this potential problem.

A limitation of this study is that we will include both prevalent and incident patients on PD. Although a study targeting only incident patients on PD might provide more solid results, we will adjust the dialysis vintage in the multivariable analyses.

In summary, the COMPASS study is the first prospective, randomized controlled trial to evaluate the clinical usefulness of BIS-guided fluid management in patients on PD. The aim of this study is to evaluate whether BIS-guided fluid management has a beneficial effect on the decline of RRF, cardiac parameters, and cardiovascular outcomes.

## Trial status

This trial is ongoing. Participants are currently being recruited.

## Abbreviations

BP: Blood pressure
BIS: Bioimpedance spectroscopy
BWT: Body weight
CBC: Complete blood count
Cr: Creatinine
ECW: Extracellular water
GFR: Glomerular filtration rate
HD: Hemodialysis
hs-CRP: High-sensitivity C-reactive protein
ICW: Intracellular water
ITT: Intention-to treat
OH: Overhydration
PD: Peritoneal dialysis
PP: Per-protocol
PWV: Pulse wave velocity
RRF: Residual renal function
TBW: Total body water.
